# Exploring gut microbiota profile induced by antipsychotics in schizophrenic patients: insights from an Eastern European pilot study

**DOI:** 10.1186/s12888-025-07461-4

**Published:** 2025-10-30

**Authors:** Ilinca-Bianca Nita, Irina-Cezara Văcărean-Trandafir, Roxana-Maria Amărandi, Ovidiu-Dumitru Ilie, Petru-Romeo Dobrin, Andreea-Cristina Bejenariu, Iuliu-Cristian Ivanov, Bogdan Doroftei

**Affiliations:** 1 Department of Psychiatry, Faculty of Medicine, “Grigore T. Popa” University of Medicine and Pharmacy, 16 University Street, Iasi, 700115 Romania; 2 TRANSCEND Research Centre, Regional Institute of Oncology, 2-4 General Henri Mathias Berthelot Street, Iasi, 700483 Romania; 3 Technical University of Sofia, Kliment Ohridski Boulevard 8, Sofia, 1756 Bulgaria; 4 Regional Center of Advanced Research for Emerging Diseases, Zoonoses and Food Safety, Department of Public Health, Faculty of Veterinary Medicine, “Ion Ionescu de la Brad” Iasi University of Life Sciences, 8 Mihail Sadoveanu Alley, Iasi, 700490 Romania; 5 “Socola” Institute of Psychiatry, 36 Bucium Road, Iasi, 700282 Romania; 6 Advanced Research-Development Center in Experimental Medicine “Prof. Ostin C. Mungiu” CEMEX, “Grigore T. Popa” University of Medicine and Pharmacy, 16 University Street, Iasi, 700115 Romania; 7 Department of Mother and Child, Faculty of Medicine, “Grigore T. Popa” University of Medicine and Pharmacy, 16 University Street, Iasi, 700115 Romania; 8 “Cuza Voda” Clinical Hospital of Obstetrics and Gynecology, 34 Cuza Voda Street, Iasi, 700038 Romania; 9 Origyn Fertility Center, 3C Palace Street, Iasi, 700032 Romania

**Keywords:** Schizophrenia, Gut microbiome, Antipsychotics, Amplicon sequencing, MiSeq, 16S rRNA

## Abstract

**Background:**

Schizophrenia (SCZ) is an intricate multi-systemic mental illness which has been linked to alterations of the gastrointestinal (GI) microbiota via the bidirectional network known as the gut-brain axis (GBA). However, the specific microbial signatures characterizing SCZ remain unclear.

**Methods:**

We analyzed the gut microbiome composition of 57 Romanian adults, 30 SCZ, and 27 healthy controls (HCs), using amplicon sequencing on the V3-V4 region of the 16S rRNA gene isolated from stool samples. DNA was extracted with an optimized enzymatic and mechanical lysis protocol. Sequence profiling was performed using DADA2. Counts were variance‑stabilized (DESeq2 VST) for Bray–Curtis and weighted UniFrac distance calculation, principal coordinate analysis (PCoA), and permutational multivariate analysis of variance (PERMANOVA). To account for data compositionality, counts were also centered log‑ratio (CLR)–transformed for principal component analysis (PCA) and supervised sparse partial least square linear discriminant analysis (sPLS‑DA). Differential abundance was tested using Wilcoxon rank‑sum (false discovery rate - FDR corrected) and linear discriminant analysis (LDA) effect size (LEfSe) (LDA > 4, FDR < 0.01).

**Results:**

SCZ patients exhibited decreased *Bifidobacterium* (*p* = 0.0187), *Blautia* (*p < 0.001*), and *Eubacterium* (*p* = 0.00120) abundances compared to HCs. Additionally, SCZ patients showed higher Faith’s phylogenetic diversity both before (*p* = 0.049) and after rarefaction (*p* = 0.0022), while Shannon and Simpson indices were not significantly different between the two study groups. PCoA on Bray-Curtis and weighted UniFrac distances revealed clear SCZ-HC separation, with unadjusted PERMANOVA attributing 13–15% of variance to SCZ status alone (*p* = 0.001). After adjusting for smoking, diet, lifestyle, metabolic and comorbidity covariates, SCZ status remained the only significant term which contributed to variance in microbiome composition (R^2^ ~ 4.4%, *p* = 0.001). Within the SCZ group, only risperidone use (not age, sex, body mass index - BMI, glycaemia, lipids, or other medication use) was significantly associated with community structure (*p* < 0.01). PCA and sPLS-DA revealed that the principal drivers for group separation after taking into account the compositional nature of data were *Erysipelotrichaceae UCG-003* and *Anaerostipes* in HCs and *Holdemanella* in SCZ (all *p* < 0.0001).

**Conclusions:**

Our integrative analyses provide evidence of gut microbiome alterations in SCZ and reveal that risperidone use modulates community structure, underscoring the need to consider the GI effects of antipsychotics when choosing appropriate regimens for patients, while at the same time highlight the GBA as a promising target for future therapeutic strategies.

## Introduction

SCZ is a complex neuropsychiatric disorder [1–3] that affects approximately 1% of the global population, exceeding 24 million diagnosed cases. According to the 2019 Global Burden of Disease Study (GBD), SCZ ranks 20th among all causes of years lived with disability (YLD) and 3rd when considering the spectrum of mental disorders. Based on the latest statistics, Romania’s age-standardized disability-adjusted life years (DALY) rate for SCZ in 2021 was estimated at 166.7 per 100,000 population compared to a European Union (EU) average of 194.0. However, most mental health burden estimates for Romania, including those reported by the World Health Organization (WHO), rely on routine data from primary care and family physicians, rather than large-scale, population-based epidemiological surveys. This reliance may contribute to the underestimation of the true prevalence and burden of SCZ. Despite the availability of modelled burden estimates, there remains a notable lack of up-to-date, SCZ-specific prevalence data from Romania, underscoring the urgent need for targeted epidemiological research [4].

SCZ patients exhibit a short life expectancy [5, 6], typically between 10 and 15 years, due to SCZ-associated comorbidities, which otherwise correlate to the elevated relapse and mortality rates [7, 8]. Consequently, SCZ substantially burdens public health systems and is associated with a progressive decline in the quality of life (QoL) [4, 9, 10]. The initial clinical signs of SCZ typically emerge in late adolescence or early adulthood [11, 12], with a higher incidence in males [13] and onset of a 3–5 year prodromal phase [14]. Its clinical phenotype spans multiple dimensions, including mood and neurocognitive features, independently from the positive and negative symptoms [2]. Alongside the widely accepted gene-environment interaction model [15], multiple hypotheses have been proposed to clarify SCZ etiology [16–18], with age identified as a contributing factor [9, 19]. Environmental influences such as infections, childbirth, and psychosocial events may act independently or synergistically in the pathobiology of SCZ [20]. Conversely, genetic predisposition accounts for only a small proportion of SCZ susceptibility [21–23]. Although treatment strategies have evolved, antipsychotic medications remain the first-line therapy in SCZ. However, 30–40% of patients do not respond adequately, and many discontinue treatment due to side effects [24], especially GI issues [25]. While these medications acting primarily as dopamine antagonists [26] are effective in reducing positive symptoms, they offer limited benefit for negative symptoms and cognitive deficits [27, 28] despite ongoing advances [29–31]. Clinical response to antipsychotics varies considerably [3, 32], and up to one-third of patients may be non-adherent [33], even with sufficient dopaminergic receptor blockage. This often results in persistent symptoms that negatively affect their QoL [25, 34]. While antipsychotics can diminish overall symptoms, approximately 60% of patients [35] experience side effects [24], a frequency that is double that in healthy individuals [36]. These limitations underscore the urgent need for novel therapeutic strategies that go beyond dopaminergic targets, particularly for treatment-resistant cases [37].

Despite extensive research focused on the pathophysiology of SCZ, recent advances in the field of genomics have set new trajectories for exploring the potential role of gut-resident microorganisms in the progression and severity of SCZ [15, 38], particularly in relation to neuroinflammation processes [39, 40]. The human gut microbiome is increasingly recognized as an essential metabolic organ facilitating energy extraction from dietary components and supplies essential nutrients to the host [41]. Comprising an estimated 150 to 400 unique bacterial species [42], this vast microbial ecosystem actively influences brain development [43] and function through various signaling molecules [44–46] referred to as the brain-immune-gut axis [47, 48].

Recent findings indicate that gut dysbiosis in the pathophysiology of SCZ is mediated by the bidirectional relationship known as the GBA. This complex network includes immune, neural, endocrine, and metabolic systems [49–51], enabling the crosstalk between the gut microbiota and the central nervous system (CNS) via several pathways [52, 53]. Both preclinical and clinical studies have consistently demonstrated that SCZ patients show changes in the gut microbial composition. These bacterial shifts highlight elevated amounts of lactic acid-producing and glutamate/gamma-aminobutyric acid (GABA)-related bacteria, alongside a notable decrease in short-chain fatty acid (SCFA)-producing species [54, 55]. Such microbial imbalances can trigger systemic inflammation, impair the blood-brain barrier (BBB) integrity, and disrupt neurotransmitter signaling, all of which are linked to the neurobiological mechanisms underlying SCZ [56–58].

Recent meta-analyses indicate that SCZ and antipsychotic treatment strategies significantly influence the gut microbiota composition, revealing overlapping as well as distinct microbial alterations. Notably, patients treated with antipsychotics display a reduced α- and increased β-diversity [59]. In contrast, drug-naïve individuals may exhibit an elevated α-diversity, suggesting a potential normalization effect during the early stages of treatment. Certain bacterial genera show consistent alterations across groups with SCZ and those undergoing medication, pointing to the possibility of shared microbial markers. Furthermore, changes in the gut microbiota caused by antipsychotics may contribute to metabolic side effects associated with second-generation antipsychotics (SGAs) [60]. These findings highlight the potential role of gut microbiota in mediating therapeutic responses. Collectively, the evidence supports the increasing interest in microbiome-targeted interventions aimed at enhancing efficacy and minimizing adverse effects in SCZ management [61].

Despite these advances, the precise mechanisms linking gut dysbiosis to CNS dysfunction are not completely understood, emphasizing the need for comprehensive studies incorporating microbiome profiling. Therefore, the primary objective of this pilot study is to investigate differences in gut microbiota composition between Romanian SCZ patients and HCs. This approach may lead to additional research to address current gaps and improve our understanding of region-specific microbial signatures while contributing to international efforts that routinely integrate such analyses.

## Methods

### Study design and participants

#### Ethical approval

This research was conducted in accordance with the principles outlined in the Declaration of Helsinki regarding Human Rights, as well as other relevant National and European regulations governing biomedical research. This study protocol received ethical approval from the Ethics Committee of the “Socola” Institute of Psychiatry (no. 7523/7.03.2023) and from “Grigore T. Popa” University of Medicine and Pharmacy in Iasi (no. 404/28.02.2024). All HCs participated voluntarily and provided written informed consent, whereas SCZ patients underwent evaluation by their psychiatrist before enrollment to confirm their eligibility for participation. If the research team unanimously agreed that a patient was unable to complete the symptom assessment measurements or fully understand the purpose of the study, that individual was excluded from participation. Participant confidentiality was maintained at all times.

#### Participants

This research included 89 participants (37 men:52 women). Among these, 40 participants (10 males:30 females) were HCs recruited from the same local community and interviewed during standard laboratory test analyses. The SCZ group comprised 49 patients (27 males:22 females). The diagnosis of SCZ was made according to the International Classification of Diseases-10 (ICD-10) as they are chronic patients with a history of SCZ and regulatory institutions from Romania adhere with the current standards established by WHO. Patients were recruited from the outer sections of Bârnova and Șipote, which serve as tertiary external pavilions of the “Socola” Institute of Psychiatry from March to August 2023. Patient data, including their updated anthropometric measurements and metabolic parameters, were collected by reviewing the medical records.

#### Inclusion and exclusion criteria

All participants were Romanian residents from the Moldova region, and reported no specific religious beliefs. Individuals from both groups were required to be 18 years of age or older. Additionally, SCZ patients were required to have maintained a stable psychopharmacological treatment regimen over the previous six months, with no dosage changes exceeding 25%. Conversely, those that met one or multiple criteria were automatically excluded from the study: (1) alcohol and/or illicit substances dependence up for the last 6 months, except of nicotine and caffeine, (2) regime based on syn-, pre- and pro-, antibiotics, or anti-inflammatory medication in the previous month, (3) history of a bacterial, fungal or viral infection, (4) severe GI symptoms (*e.g*. diarrhea or constipation) in the previous month, and (5) history of systemic diseases or other comorbidities that could disrupt gut microbiota stability in the last 3 months, and (6) severely unbalanced diet or changes in eating behavior. HCs underwent thorough clinical assessments, including physical examinations and laboratory tests to exclude medical or psychiatric conditions, adhering to the same inclusion and exclusion criteria as SCZ patients, except for a SCZ diagnosis.

Of the initial cohort, 19 participants (21.34%) were excluded from the study because of missing clinical data, failure to meet the inclusion criteria, or inability to provide a fecal sample. Given the study design, we opted for a complete-case analysis rather than imputing clinical covariates; this approach preserves analytic simplicity but reduces statistical power and may introduce bias if exclusions are non-random [62]. The final datased comprised 70 subjects (*n* = 35 HCs [10 male:25 female] and *n* = 35 SCZ [18 male:17 female]) (28 male:42 female). SCZ patients were additionally evaluated using the Brief Psychiatric Rating Scale (BPRS) [63, 64]. Most SCZ patients were prescribed antipsychotic medications, including both first (*n* = 3 – Haloperidol, *n* = 1 – Levomepromazine) or SGAs, which included (*n* = 14 – Olanzapine, *n* = 6 – Risperidone, *n* = 2 – Quetiapine, *n* = 1 – Clozapine, *n* = 1 – Aripiprazole, *n* = 1 – Paliperidone, and *n* = 1 – Amisulpride). Patient comorbidities according to ICD-10 were grouped in six main categories: metabolic (E11.x, E66.x, E78.x), cardiovascular (I10-I15, I20-I25, I30-I52, I70-I79), hepatobiliary (K70-K76, K80-K82, B18.x), renal/urinary (N00-N08, N10-N29, N30-N40), pulmonary (J40–J47, J80–J99) and other conditions (all remaining codes).

### Sample processing and sequencing

#### Sample collection

To prevent degradation of the genetic material, fecal samples were initially collected in sterile plastic containers and stored at -20 °C within the first 15–30 min. This protocol aligns with previous studies indicating that short-term storage up to 24 h, or longer does not significantly affect microbial composition when samples are kept at appropriate temperatures, with or without the use of preservative buffers [65–68]. Subsequently, all stool samples were transported to the laboratory on dry ice and stored at -80 °C until further processing. In line with the collection timeline, the entire batch was processed within one year, a timeframe during which fecal samples are considered to remain microbiologically stable, with no significant alterations in microbial structure, composition, or diversity [69].

#### DNA extraction procedure

DNA extraction was optimized and conducted in batches of 12 samples to minimize potential cross-contamination. DNA was isolated from ~ 350 mg fecal material using the NucleoSpin^®^ Soil kit (Macherey-Nagel, Düren, Germany) according to the manufacturer’s instructions with additional steps. The NucleoSpin^®^ Soil protocol was followed with lysis buffer SL2 and Enhancer SX, as previously described [70, 71].

#### Enzymatic and mechanical lysis

To optimize the breakdown of both gram-positive and gram-negative bacteria [72], lysis buffers, incubation times, and enzyme concentrations were tested using both SL1 and SL2 buffers from the NucleoSpin^®^ Soil kit, proteinase K (Thermo Fisher Scientific, Massachusetts, US), lysozyme, and lysostaphin (Sigma-Aldrich Co., US) (data not shown). The enzymatic lysis process was refined for optimal results by incubating samples overnight at 37 °C with 800 µL of SL2 and 40 µL of proteinase K (20 mg/mL ~ 600U/mL). This was followed by an additional 1-hour incubation at 37 °C with 50 µL of lysozyme (10 mg/mL ~ 40 KU/mL) and 3 µL of lysostaphin (1 mg/mL ~ 3 KU/mL). After adding each reagent, the samples were vortexed vigorously for a few minutes.

After enzymatic lysis, mechanical lysis was conducted using a FastPrep-24™ homogenizer (MP Biomedicals) at a speed of 6 m/sec for 80 s with MN Bead Tubes type A from the NucleoSpin^®^ Soil kit that contains ceramic beads. Two centrifugation steps were performed for 2 min at 11,000 x *g*, as per the manufacturer’s protocol, to generate a clear supernatant after the fecal material was lysed. Following chemical and mechanical lysis, roughly 700 µL of the supernatant was transferred to a NucleoSpin^®^ inhibitor removal column for the binding, washing, and elution processes. After heating the SE elution buffer to 80 °C, a final eluate containing 30 µL of genomic DNA (gDNA) was obtained, then quantified and stored at -20 °C until use. Negative controls were included in every DNA extraction, where the appropriate buffer was used without adding any fecal material.

#### Assessment and measurement of extracted DNA

DNA concentration (A260) and purity (A260/230 and A260/280 ratios) were measured using a NanoDrop spectrophotometer (Thermo Fisher Scientific, Massachusetts, US). Ratios from 1.8 to 2.0 indicated low protein contamination while A260/230 ratios assessed contamination with organic compounds/phenols or carbohydrates. DNA integrity and size were verified by gel electrophoresis on a 2% agarose gel stained with ethidium bromide, run in 1x TAE buffer at 180 V.

#### 16S V3-V4 rRNA sequencing

Following Illumina’s guidelines, a 16S metagenomic sequencing library was created in order to identify the bacterial makeup of each sample [73, 74]. In summary, we used primer paired sequences with overhang adapters to target the V3-V4 region of the 16S rRNA gene in the samples. This first PCR produced an amplicon of about ~ 460 bp [74]. The following thermal cycling conditions were used in a final volume of 25 µL: 12.5 µL 2X KAPA HiFi HotStart Ready Mix (Roche Holding AG, Basel, Switzerland), 2.5 µL of microbial genomic DNA and 5 µL of each forward and reverse 1 mM primer with the following thermal cycling conditions: denaturation for 3 min at 95 °C, followed by 25 cycles for 30 s at 95 °C, 55 °C, and 72 °C, and a closing extension step for 5 min at 72 °C. After that, the sequences were purified using Agencourt AMPure XP magnetic beads (Beckman Coulter, Brea, US) in order to remove primer dimer species or excess primers, nucleotides, salts, and enzymes on a BIOMEK^®^ FXP automatic workstation (Beckman Coulter, Brea, US). Following this step, a second PCR was conducted from 5 µL of the purified PCR amplicons to add the dual indexes and sequencing adapters with the Nextera XT Index Kit V2 set A (Illumina, San Diego, US) with 5 µL of Nextera XT Index 1 Primers F (10 M) and Nextera XT Index 2 Primers R (10 M) each, 25 µL of 2X KAPA HiFi HotStart Ready Mix (Roche Holding AG, Basel, Switzerland), and 10 µL ddH2O in a final reaction volume of 50 µL. After three minutes of activation and denaturation at 95 °C, eight cycles of 30 s each at 95 °C, 55 °C, and 72 °C completed the thermal cycling program, with an ending elongation step of 5 min at 72 °C. The final library was cleaned up using AMPure XP magnetic beads (Beckman Coulter, Brea, US) in a second purification before quantification. DNase-free water was used as a negative control during library preparation. The Qubit 4 fluorometer and the Qubit 1X dsDNA High Sensitivity Assay Kit (Thermo Fisher Scientific, Massachusetts, US) were used to measure the DNA concentration of the PCR products. To create a 4 nM library, the barcoded amplicon libraries were combined in equimolar amounts. A final concentration of the pooled samples of 12 pM with 20% Phix control (Illumina, San Diego, US) was used to sequence the V3–V4 region of the 16S rRNA gene libraries on the Illumina MiSeq platform using a paired-end (2 × 300 bp (PE300)) sequencing kit.

### Bioinformatics and statistical analysis

#### Sequence pre-processing and denoising

All analyses were performed in R (version 4.3.2). The DADA2 pipeline [75] was followed to process demultiplexed sequences, similar to what has been previously described [70, 71]. Following primer removal, forward/reverse sequences were trimmed to 255/215 bases; reads with ambiguous bases were discarded. Paired reads were merged, chimeras were detected and removed, and only merged sequences >350 bases were retained. Taxonomy was assigned using the RDP Naive Bayesian classifier implemented in dada2 v 1.30.0, against the database SILVA 138 (80% bootstrap confidence) [76, 77]. Phylum level-unclassified amplicon sequence variants (ASVs), as well non-bacterial sequences were discarded. Samples with fewer than 5,000 reads were excluded (*n* = 13), leaving 57 samples in the study, (27 HCs and 30 SCZ patients). Resulting ASV tables were used for generating phyloseq objects (phyloseq package v 1.46 [78]). Sequences classified as the same species or sequences unclassified at the species level were agglomerated.

#### Bacterial composition and diversity analysis

Genus-level counts were converted to relative abundances for descriptive barplots (generated using ggplot2 v 3.5.1 [79]). Observed species, Shannon and Inverse Simpson indices were calculated on both full count data and rarefied counts (5,171 reads/sample) using the vegan package v 2.6-4 [80], while Faith’s phylogenetical distance (Faith’s PD) was computed using picante package v 1.8.2 [81]. Count tables were first normalized using the variance stabilizing transformation (VST) in DESeq2 v 1.42.1 [82], to correct for unequal library sizes while avoiding the information loss usually associated with rarefaction [83]. Bray‒Curtis distances (phyloseq) and weighted UniFrac distances (GUniFrac package v 1.8 [84]) were then computed from VST-normalized counts, visualized by PCoA, and compared between groups using PERMANOVA with 999 permutations, with dispersion checked via betadisper (vegan). Both univariate and multivariate PERMANOVA after correcting for potential confounders were performed.

Because microbiome data are inherently compositional [85], all downstream multivariate and supervised analyses were performed on CLR-transformed abundances. PCA and sPLS-DA were performed using mixOmics v 6.26.0 [86], and the sPLS-DA model was evaluated using 5-fold cross-validation repeated 50 times.

#### Statistical analysis

Continuous features were tested for normality using the Shapiro-Wilk test. Normally distributed variables were compared with two-sample *t*-test; non-normally distributed variables were compared by Wilcoxon rank-sum test. All multiple comparisons were corrected using the Benjamini–Hochberg (BH) FDR procedure, with an FDR threshold of 0.05, unless otherwise stated. Categorical variables were compared by Pearson’s chi-squared or Fisher’s exact test when any cell count was < 5.

Genus-level relative abundances and α-diversity indices were tested by Wilcoxon rank-sum with FDR correction. LEfSe, as implemented in the microbiomeMarker package v 1.8.0 [87], was used for assessing differentially abundant taxa between groups from genus-agglomerated phyloseq objects, using an LDA score cutoff of 4 and an FDR-adjusted Kruskal-Wallis *p*-value cutoff of 0.01.

Multicollinearity among sample features was assessed via variance inflation factors (VIFs), calculated using the car package v 3.1-3. Only variables with VIF < 4 were retained for model building.

Post-hoc power calculations for key effect sizes were performed using the pwr package v1.3-0.

## Results

### Anthropometric and metabolic parameters

Laboratory test results for both enrolled groups (Table 1) revealed notable differences across various parameters. SCZ patients were on average shorter (*p* = 0.008) and lighter (*p* < 0.001) than HCs, yet their BMI did not differ significantly (*p* = 0.073), indicating comparable overall adiposity. Fasting glycemia, total cholesterol, and LDL-cholesterol were significantly different between groups (*p* < 0.001), suggesting these measures could act as confounders in subsequent analyses of microbial composition. In contrast, gender, age, BMI, HDL-cholesterol, and triglyceride levels showed no significant differences between groups (*p* >0.05). However, we observed a striking difference in smoking prevalence between groups (88.6% of SCZ patients vs. 11.4% of HCs; χ^2^ = 48.4, *p* < 0.001), consistent with the known higher smoking rates in SCZ [88]. In addition, HCs almost exclusively followed a common diet, whereas SCZ patients more often followed hepatic or hyposodic regimens (Fisher’s exact test, *p* < 0.001). A “common” diet in our cohort reflected unrestricted, habitual food choices, while the hepatic plan emphasized low total and saturated fats, lean protein sources, complex carbohydrates and fiber, minimal simple sugars, and complete alcohol avoidance designed to reduce hepatic metabolic load and support bile flow. The two groups also differed in overall activity patterns (Fisher’s exact test, *p* < 0.021), with SCZ patients being more likely to report a sedentary lifestyle (88.6% SCZ vs. 62.9% HCs). In addition, several organ-system comorbidities were significantly more common in SCZ than in HCs (Fisher’s exact tests): cardiovascular disease (*p* = 0.004), hepatobiliary disorders (*p* < 0.001), renal/urinary disorders (*p* < 0.001), as well as other disorders (*p* = 0.0125). By contrast, metabolic and pulmonary comorbidities did not differ between groups (*p* >0.05).

**Table 1:** Demographic, anthropometric, metabolic, and confounder information of the study participants

		Entire group (*N* = 70)	HC (*N* = 35)	SCZ (*N* = 35)	*p* value	Observations
Gender	M: F	28:42	10:25	18:17	0.0876	Chi-squared test
Age (Years)	M (SD)	57.27 (11.94)	54.91 (13.75)	59.62 (9.43)	0.0997	t-test
	Range	20–80	20–76	40–80		
Height (cm)	M (SD)	168.17 (7.06)	170.37 (5.81)	165.97 (7.58)	0.0083	t-test
	Range	150–186	152–186	150–180		
Weight (kg)	M (SD)	68.65 (9.76)	72.45 (8.26)	64.85 (9.77)	< 0.001	t-test
	Range	45–89	50–89	45–89		
BMI	M (SD)	24.27 (3.23)	24.96 (2.56)	23.57 (3.70)	0.0732	t-test
	Range	16.33–35.65	18.5–29.4	16.33–35.65		
Glycemia	M (SD)	95.98 (27.02)	100.74 (34.94)	91.22 (14.62)	< 0.001	Wilcoxon rank-sum test
	Range	48–272	48–272	70–130		
Cholesterol	M (SD)	190.47 (46.92)	212.25 (50.25)	168.68 (31.09)	< 0.001	Wilcoxon rank-sum test
	Range	114–321	114–321	122–275		
HDL	M (SD)	53.67 (14.11)	56.54 (14.87)	50.81 (12.89)	0.0896	t-test
	Range	30–90	30–90	30–73		
LDL	M (SD)	117.37 (44.98)	141.82 (48.29)	92.91 (23.31)	< 0.001	Wilcoxon rank-sum test
	Range	62–263	65–263	62–179		
Triglycerides	M (SD)	128.22 (63.91)	140.28 (77.16)	116.16 (45.13)	0.445	Wilcoxon rank-sum test
	Range	44–339	44–339	53–276		
BPRS Score	M (SD)			55.15 (8.92)		
	Range			35–72		
Smoking	Y: N	35:35	4:31	31:4	< 0.001	Fisher’s Exact test
Diet					< 0.001	Fisher’s Exact test
	Common	42	34	8		
	Hepatic	21	0	21		
	Low sodium	2	0	4		
	Hepatic + Low sodium	4	0	2		
	Low glycemic index	1	1	0		
Lifestyle					0.021	Fisher’s Exact test
	Active	3	3	0		
	Minimal activity	14	10	4		
	Sedentary	53	22	31		
Comorbidities						
	Metabolic	9	3	6	0.4773	Fisher’s Exact test
	Cardiovascular	8	0	8	0.004	Fisher’s Exact test
	Hepatobiliary	26	1	25	< 0.001	Fisher’s Exact test
	Renal/Urinary	20	1	19	< 0.001	Fisher’s Exact test
	Pulmonary	3	0	3	0.2391	Fisher’s Exact test
	Other	18	4	14	0.0125	Fisher’s Exact test

### Bacterial composition

**Fig. 1:**
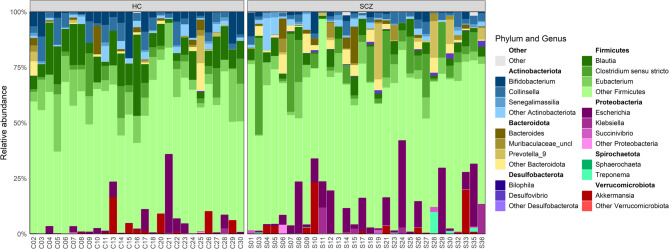
Comparative overview of dominant phylum and genus-level microbial composition in SCZ and HC individuals; corresponding phyla highlighted in bold above each genus group

After quality filtering and removal of samples with fewer than 5,000 reads, 57 participants remained (30 SCZ, 27 HCs; 25 M:32 F). 5,165,138 sequences were read across all samples (mean 73,787, median 33,060 reads/sample). Following trimming, denoising and chimera removal, 42.5% of sequences remained, yielding 387 unique bacterial sequences in the final feature table. Figure 1 shows, for each sample, the three most abundant genera within the first seven dominant phyla. Both SCZ and HC were dominated by the phyla Firmicutes and Actinobacteriota, with lower relative abundance contributions from Spirochaetota, Desulfobacterota, and Verrucomicrobiota. However, SCZ samples showed a clear relative enrichment of Proteobacteria and Bacteroidota compared to HCs, suggesting a shift in gut-microbial balance associated with disease status.

**Fig. 2:**
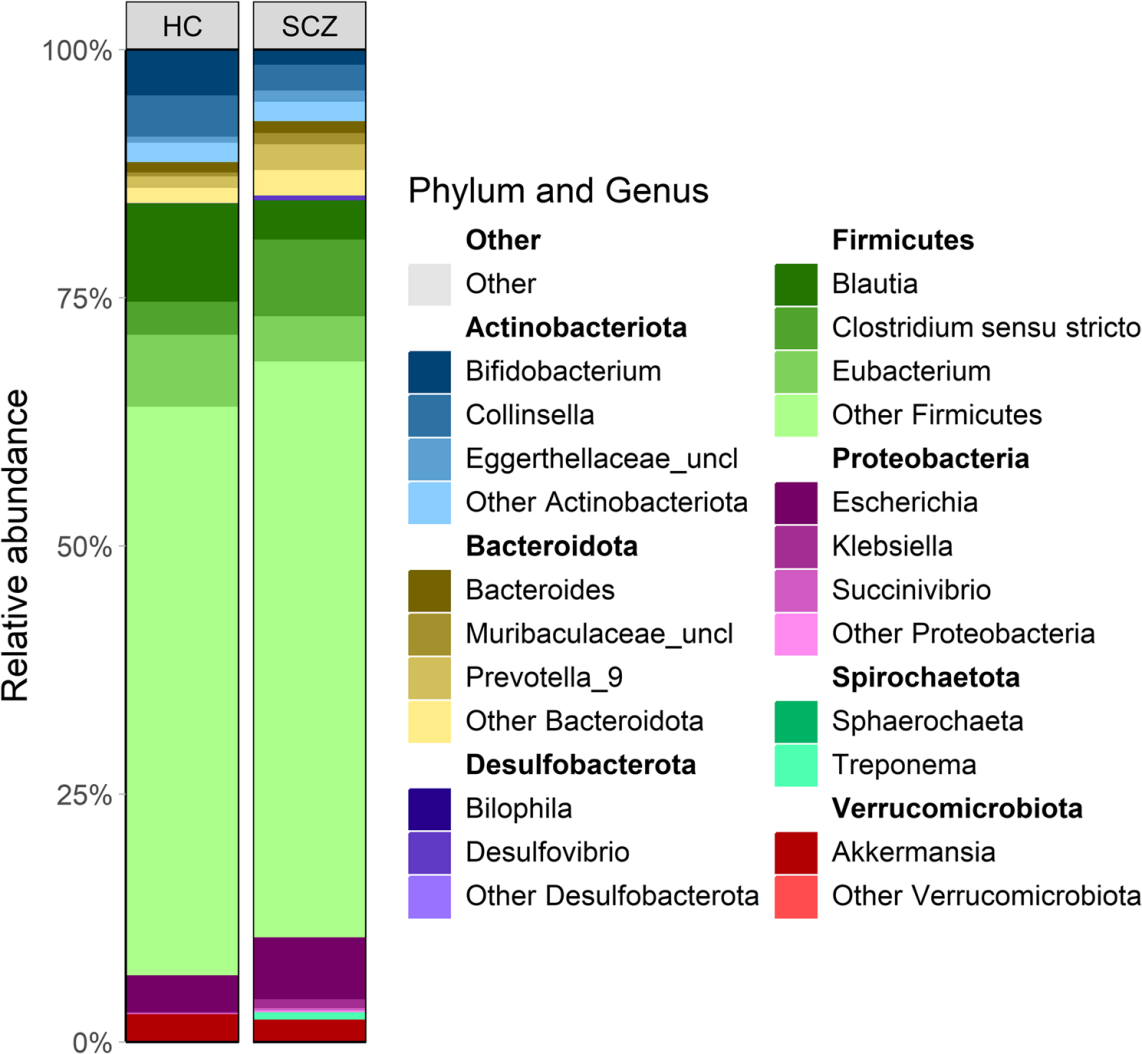
Bacterial community structure in terms of relative abundances merged at genus level per each group in SCZ and HC individuals

When comparing mean genus-level relative abundances between SCZ and HCs, we found a significant depletion of *Bifidobacterium* (1.6 ± 0.48% in SCZ vs. 4.97 ± 1.01% in HC, FDR-adjusted *p* = 0.0187), *Blautia* (3.77 ± 0.49% in SCZ vs. 11.08 ± 1.45% in HC, FDR-adjusted *p* = 0.000341), and *Eubacterium* (4.26 ± 0.73% in SCZ vs. 9.74 ± 1.13% in HC, FDR-adjusted *p* = 0.00120) in the SCZ group. In addition, some bacteria belonging to the phyla Spirochaetota and Desulfobacterota were detected exclusively in SCZ patients (Fig. 2).

### Bacterial diversity

#### α-diversity

**Fig. 3:**
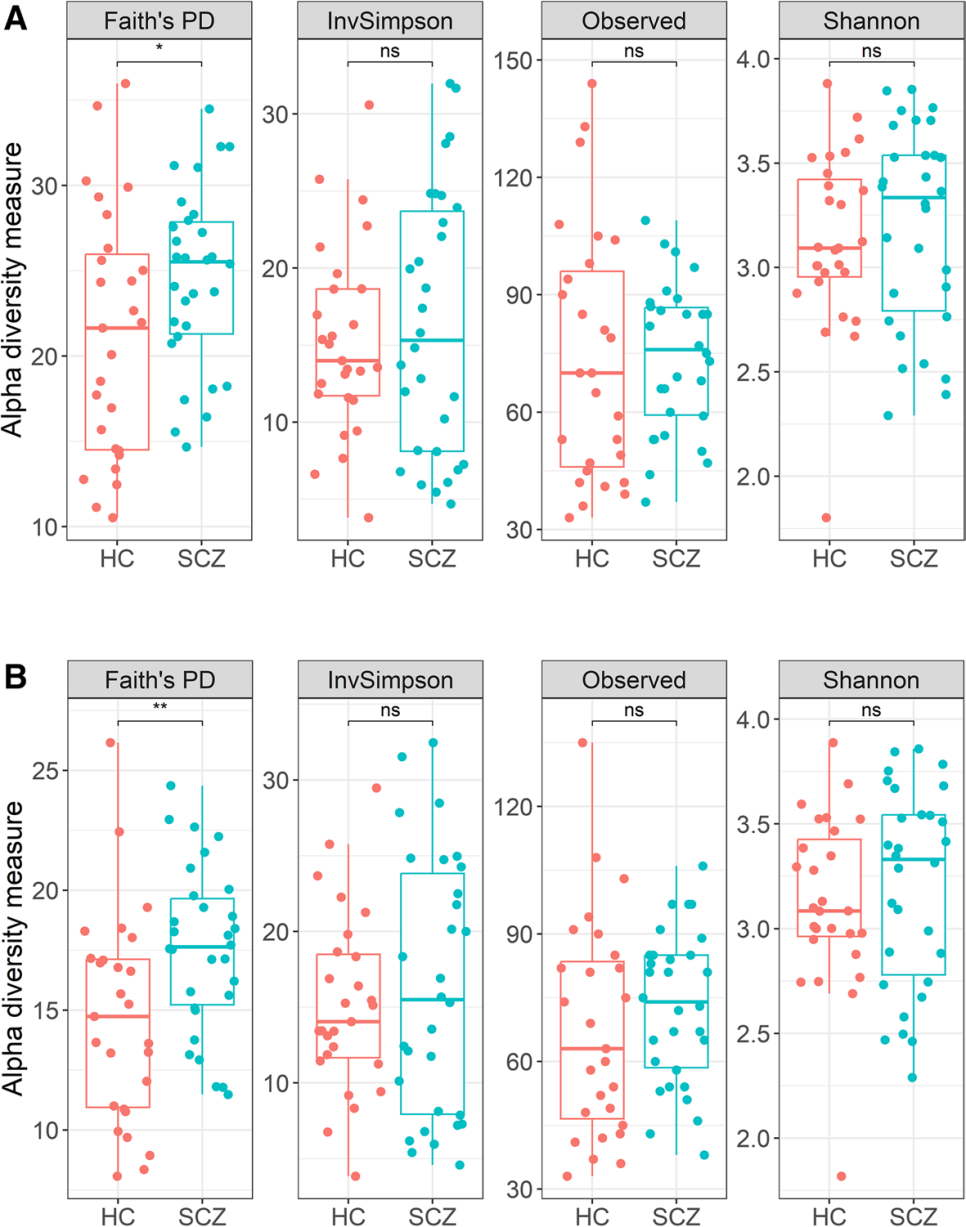
Alpha diversity metrics: Faith’s phylogenetic diversity, inverse Simpson index, observed number of unique sequences and Shannon index on (**A**) all sequences; (**B**) after rarefaction; * *p* < 0.05, ** *p* < 0.01; ns – not significant (FDR-adjusted *p*, Wilcoxon rank-sum test)

We observed a statistically significant difference in Faith’s PD between SCZ and HC groups (Wilcoxon rank-sum test, FDR-corrected, *p* = 0.049), with other α-diversity metrics (Inverse Simpson, Observed Richness, Shannon index) showing no significant differences between groups (Fig. 3A).

Since variation in sequencing depth can bias α-diversity metrics [89], we rarefied all samples to 5,171 reads (the smallest library size in our dataset). The significant difference in Faith’s PD is preserved after rarefaction (Fig. 3B, *p* = 0.0022), confirming that SCZ patients harbor greater phylogenetic diversity than of HCs, in line with what others have found [90].

#### β-diversity

We began our analysis with PCoA and PERMANOVA on Bray-Curtis and weighted UniFrac distances computed from VST-transformed counts to visualize broad community shifts, as per our previous protocols [70, 71].

**Fig. 4:**
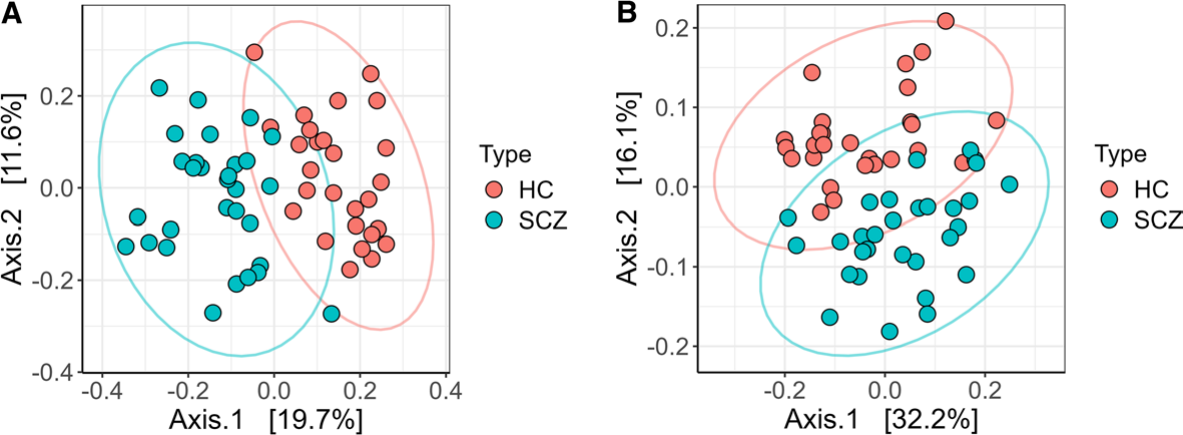
PCoA analysis based on (**A**) Bray–Curtis dissimilarity matrix after VST transformation of raw abundances and (**B**) weighted Unifrac distance after VST transformation of raw abundances. Colors represent sample types: red – HC; blue – SCZ; ellipses are drawn at 95% confidence level. The percentage of variation explained by the first two dimensions is indicated on respective axes

PCoA analysis on the Bray-Curtis dissimilarity matrix showed that 31.3% of the total variance could be captured by the first two axes (Principal component 1 - PC1 = 19.7%, PC2 = 11.6%; Fig. 4A), while on weighted Unifrac distances, the two principal axes captured 48.3% (PC1 = 32.2%, PC2 = 16.1%; Fig. 4B). 95% confidence ellipses illustrate a clear, though not complete, separation of the two groups, with only modest overlap.

**Fig. 5:**
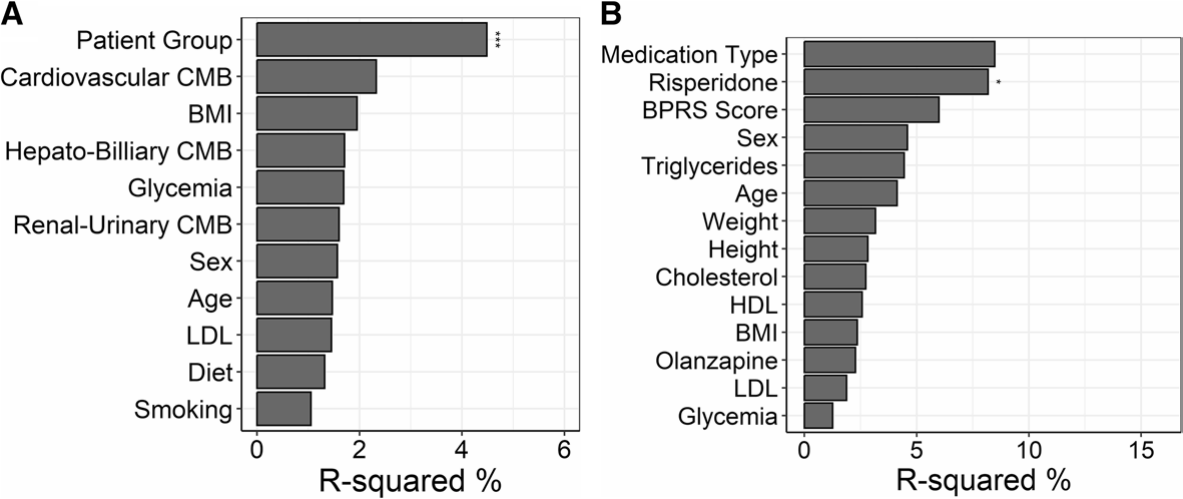
Adonis PERMANOVA comparison with metadata for (**A**) entire study population and (**B**) only SCZ patients; R-squared refers to amount of Bray-Curtis variation associated with each category; *** *p* < 0.001; * *p* < 0.05

We performed both univariate and multivariate PERMANOVA adjusting for potential confounders to assess whether disease status drives gut microbiome variation independently of these clinical characteristics. Since fasting glycaemia, total cholesterol and LDL-cholesterol were the metabolic parameters which differed significantly between groups (all *p* < 0.001; Table 1), and given that total cholesterol and LDL were moderately colinear (VIF ≈ 4), we selected LDL as our lipid covariate and omitted total cholesterol to avoid redundancy. Additional co-variates included in the analysis were age, sex, BMI, diet, smoking status, lifestyle and comorbidities. In the univariate PERMANOVA on Bray-Curtis distances, SCZ status alone explained 14.72% of the variance (pseudo-F = 9.49, *p* = 0.001). After adjusting for potential confounders, SCZ vs. HCs still accounted for 4.37% of the community-composition variance (pseudo-F = 2.83, *p* = 0.001), retaining significance. All other clinical and lifestyle factors, though biologically plausible confounders contributed only trivially and none reach statistical significance (all *p* > 0.1, Fig. 5A). The shift from the sequential R^2^ of ~ 14% down to a marginal R^2^ of ~ 4.4% is expected: when SCZ is tested first, it captures both its own effect and any shared variance with covariates; when tested in the multivariate model, it is forced to compete with the other predictors for unique variance. The fact that SCZ status remains the only significant term under the marginal test underscores its robust, independent association with gut-microbiome composition.

A similar pattern was shown by PERMANOVA on weighted-UniFrac distances: univariate R^2^ = 13.43% (pseudo-F = 8.54, *p* = 0.001) and adjusted R^2^ = 4.49% (pseudo-F = 2.92, *p* = 0.001), with all confounders non-significant (all *p* > 0.1), highlighting the robustness of our findings. Finally, dispersion tests confirmed homogeneous dispersion across all tested parameters (*p* > 0.05), validating that these results reflect genuine shifts in gut-microbial composition rather than differences in variability. However, the fact that over 69% of variance remains unexplained suggests that additional influences such as medication, stress or genetic factors may also shape the microbiome in SCZ. Thus, we performed PERMANOVA on SCZ patients alone and found that risperidone treatment was the only significant factor which contributed to the observed variation of microbial composition (R^2^ = 8.2%, *p* < 0.05). In addition, medication type (no medication, typical antipsychotic medication, atypical antipsychotic medication or combined) accounted for 8.5% of the observed variation, but it did not have a significant contribution (Fig. 5B). Other metadata were not significantly associated with compositional variation.

**Fig. 6:**
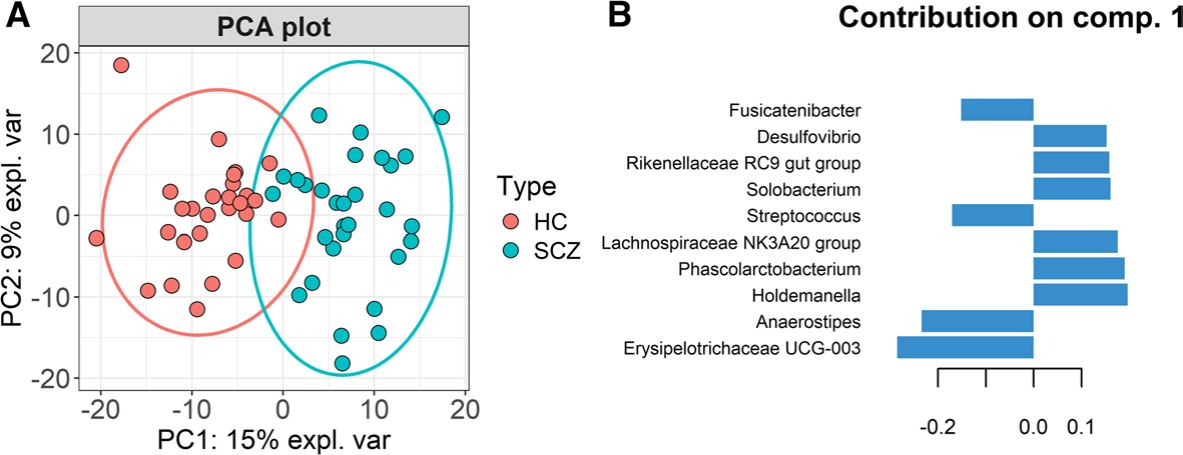
**(A**) PCA analysis starting from CLR-transformed abundance data; **(B)** Top 10 most relevant genera which contribute to PC1

PCA on CLR-transformed abundance data revealed a better separation of the two study groups based on bacterial composition (Fig. 6A). This analysis further allowed the identification of the contributions of each bacterial genus on the principal components (Fig. 6B).

Thus, the top 10 genera contributing to PC1 are *Erysipelotrichaceae UCG-003*,* Anaerostipes*,* Holdemanella*,* Phascolarctobacterium*,* Lachnospiraceae NK3A20 group*,* Streptoccocus*,* Solobacterium*,* Rikenellaceae RC9 gut group*,* Desulfovibrio* and *Fusicatenibacter*.

**Fig. 7:**
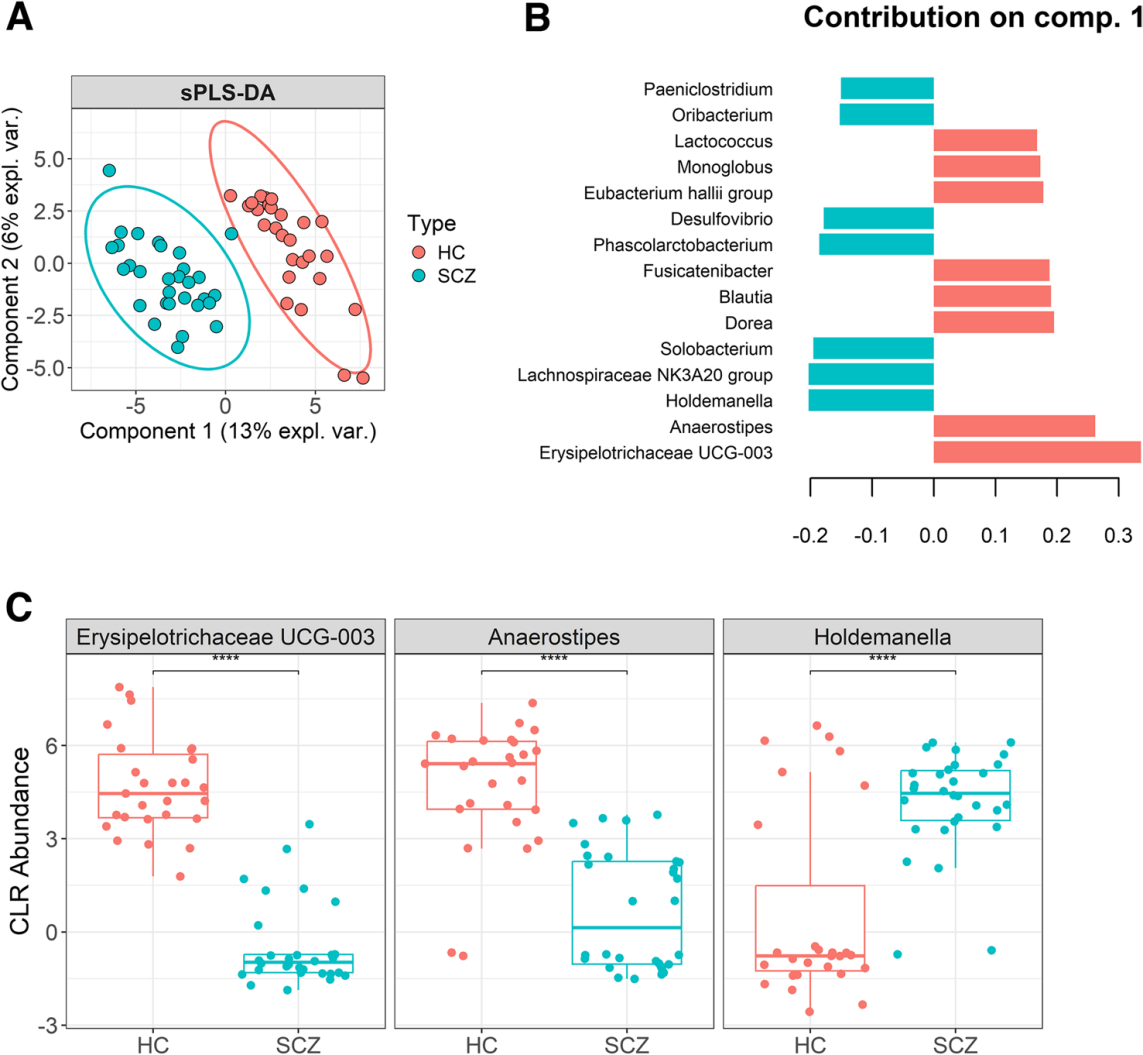
(**A**) sPLS-DA bi-plot; (**B**) Top 15 most relevant contributions for PC1 separation; (**C**) CLR abundances of the 3 most relevant genera for separation on component 1; **** *p* < 0.0001 (FDR-adjusted *p*, Wilcoxon rank-sum test)

sPLS-DA revealed a similar separation of groups based on microbial composition (Fig. 7A), albeit with some differences on component 1 contributions (Fig. 7B). The main drivers of SCZ group delimitation were the genera *Holdemanella*,* Lachnospiraceae NK3A20 group*,* Solobacterium*, *Phascolarctobacterium*, *Desulfovibrio*, *Oribacterium* and *Paeniclostridium*, while the genera *Erysipelotrichaceae UCG-003*,* Anaerostipes*,* Dorea*, *Blautia*, *Fusicatenibacter*,* Eubacterium hallii group*,* Monoglobus* and *Lactococcus* are responsible for HC group separation. Class-specific error rates from 5-fold cross-validation (×50) show that component 1 alone misclassifies only 2.9% of HC and 3.5% of SCZ samples (BER = 0.031). Adding component 2 slightly lowers the HC classification error (2.1%) but increases it for SCZ samples (7.2%), raising the BER to 0.048. Hence, a single-component model provides the best-balanced performance.

The CLR-transformed abundance of the top three most differentially abundant genera (Fig. 7C) yielded very large empirical effect sizes (smallest Cohen’s *d* = 1.49), corresponding to more than 99.8% power at *α* = 0.05. We furthermore estimated that to detect more moderate effects (*d* ≈ 0.5) with 80% power would require ∼64 subjects per group. Overall, these results demonstrate a clear differentiation between SCZ patients and HC on the basis of gut bacterial composition.

The higher abundance of *Erysipelotrichaceae UCG-003* and *Anaerostipes* in HCs suggests a possible protective role of these genera, while the higher abundance of *Holdemanella* in SCZ patients suggests the possible involvement of this genus in SCZ (*p* < 0.0001).

### Differential abundance

**Fig. 8:**
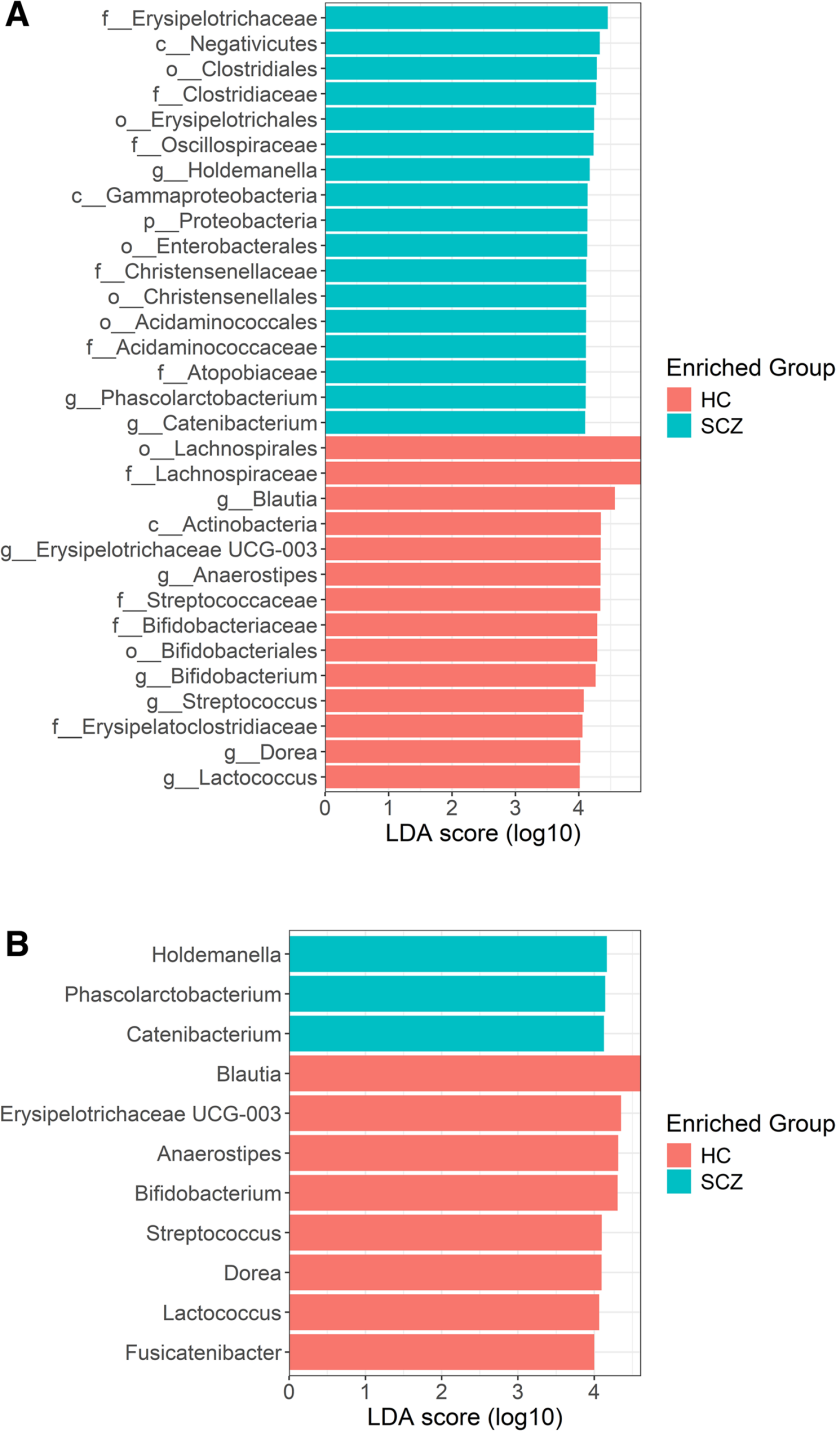
Results of differential abundance analysis by LEfSe; (**A**) at all levels: p – phylum; f – family; c – class; o – order; g – genus (**B**) only at genus level

LEfSe analysis revealed a total of 11 genera with significantly different abundance between the two groups (LDA score > 4, adjusted *p* value < 0.01, Fig. 8A). Bacteria from the genera *Holdemanella*, *Phascolarctobacterium* and *Catenibacterium* were more likely to be found in SCZ patients compared to HCs. At the family level, a higher abundance of *Erysipelotrichaceae* and *Acidaminococcaceae* was found in SCZ patients, but also of bacteria from the *Oscillospiraceae*, *Christensenellaceae* and *Clostridiaceae* families. At the order level, *Acidaminococcales*, *Christensenellales*, *Clostridiales*, *Erysipelotrichales* and *Enterobacterales* are preferentially more abundant at SCZ, and at the class level *Negativicutes* and *Gammaproteobacteria* (Fig. 8A). The genera *Blautia*, *Erysipelotrichaceae UCG-003*, *Anaerostipes*, *Bifidobacterium*, *Streptococcus*, *Dorea*, *Lactococcus* and *Fusicatenibacter* are predominantly found in HC patients (Fig. 8B).

## Discussion

The present pilot study aimed to analyze gut microbial composition of individuals diagnosed with SCZ and HCs from Romania, targeting the hypervariable region V3-V4 of the 16S rRNA gene. The phylogenetic patterns identified in our dataset are in good agreement with results from a pilot investigation involving first-episode, drug-naïve SCZ patients [91], which noted subtly similar microbial compositions among all four primary phyla in comparison to HCs. Transplanting microbiota from SCZ patients into mice can lead to pronounced phylum-level shifts, notably a reduction in Firmicutes and Bacteroidetes alongside an increase in Actinobacteria and Proteobacteria [92]. These trends are further supported by a Chinese regional cohort study, which also found elevated levels of all four phyla [93]. Nevertheless, other investigations have identified either comparable microbiota profiles between patients and controls [94], or substantiated phenotype-specific microbial alterations linked to symptom severity, immune function, and behavioral treats like aggression [95–97]. At the same time, variations in gut microbiota have been associated with the chronicity of SCZ [98] and to neuroanatomical variations tied to disease progression and antipsychotic treatment [99], which we did not account for in our own analysis.

The detected decrease in the relative abundance of *Bifidobacterium*, *Blautia* and *Eubacterium* in patients with SCZ corresponds with results from various other studies and may indicate wider disturbances, critical to host neurobiology [93, 97, 100, 101]. Numerous investigations have noted a depletion in *Bifidobacterium*, suggesting that this loss may be a feature of SCZ, defining a heightened gut permeability and peripheral immune activation [93, 100, 101]. This genus is recognized for its role in preserving intestinal barrier integrity, responsible for SCFAs production, and regulation of systematic inflammation [93, 100]. Reductions in *Blautia*, another SCFA-producing genus, have been reportedly observed in schizophrenic cohorts, including subgroups displaying aggressive behaviors [97]. This reinforces its relevance to both the core symptoms and behavioral dimensions of SCZ, thus indicating a link between microbial metabolic dysfunction and behavioral dysregulation [97, 100]. Additionally, decreases in *Eubacterium*, otherwise involved in bile acid transformation and butyrate production, processes essential for sustaining CNS homeostasis have also been recorded, further supporting the association between SCZ and a disrupted gut microbial profile [100, 101]. Despite previous studies lacking detailed quantitative comparisons, the consistency in the direction of change across various patient populations highlights a recurring pattern of microbial dysbiosis involving key genera [93, 97, 100, 101]. Isobutyric and propanoic acids are SCFAs known for their roles in enhancing gut health and regulating inflammatory responses. Both of these compounds have been negatively associated with the abundance of the *Holdemanella* genus [102], which was identified as the principal driver of SCZ delimitation in our sPLS-DA analysis. Interestingly, the abundance of *Holdemanella* has been positively linked with increased levels of certain saturated fatty acids (stearic and palmitic), along with the fatty acid byproduct monostearin in the cecum of mice [103]. Notwithstanding these connections, *Holdemanella biformis* seems to alleviate metabolic dysfunctions induced by a high-fat/sugar diet [103, 104], probably owing to its anti-inflammatory properties in colitis and its protective role against intestinal tumor development. These advantageous effects are believed to be facilitated by the long-chain fatty acid 3-hydroxyoctadecaenoic acid, a metabolite synthesized by *Holdemanella biformis* [105, 106]. In the context of SCZ, alterations in gut microbiota composition have been observed among Chinese patients with comorbid metabolic syndrome, revealing an enrichment of *Holdemanella*. Notably, the abundance of *Holdemanella* was positively correlated with the Scale for the Assessment of Negative Symptoms (SANS), suggesting a possible association between this genus and the severity of negative symptoms in SCZ [107]. Though scientific data is scarce about the presence of *Holdemanella* in the gut of SCZ patients, it has been previously associated with neuro-metabolic conditions and idiopathic GI conditions. As such, elevated levels of *Holdemanella* have been observed in obese individuals with uncontrolled eating behavior [108], diabetic patients [102], with autism spectrum disorder (ASD) [109], particularly those without constipation [110], and in Alzheimer’s disease (AD) [111], especially among those with cognitive impairment [102, 112]. Moreover, its involvement in functional gastrointestinal disorders (FGID) spectrum has been also recently documented [113, 114].

*Anaerostipes*, a key SCFA producer, was enriched in our HC group, mirroring a previous study on 36 hospitalized SCZ patients that reported significantly reduced *Anaerostipes* abundance compared to controls, indicating an impaired SCFA-producing capacity in SCZ [115]. Additionally, while a different study focusing on chronic SCZ patients did not specifically emphasize on the prevalence of *Anaerostipes*, it reported parallel microbial shifts, including reductions in potentially beneficial genera like *Clostridium* and *Sutterella* [98]. These compositional changes might furthermore intersect with host genetic and metabolic factors. For example, *Anaerostipes* is depleted in carriers of the SLC39A8 Thr391 allele among overweight or Crohn’s disease (CD) patients [116], and its levels are negatively associated with age [117] and polygenic risk scores [118].

*Erysipelotrichaceae UCG-003* was found to be elevated in HCs, compared to patients with SCZ, contradicting findings from a recent study that identified this taxon as a distinguishing microbial marker elevated in both SCZ and major depressive disorder (MDD) during hospitalization [115]. This discrepancy suggests a context-dependent role of *Erysipelotrichaceae UCG-003* in psychiatric disorders. While this genus has been observed in healthy aging population [119], it is also known to be overrepresented in inflammatory-related conditions, such as sepsis [120] and alcohol-induced chronic pancreatitis (AICP) patients [121]. Its overabundance may therefore reflect underlying low-grade inflammation or metabolic disruptions associated with psychiatric symptomatology. Interestingly, psychotropic medications have been shown to reduce abundance of *Erysipelotrichaceae* overall [122], and this reduction is consistent with observations in individuals with ASD relative to HCs [123]. These conflicting patterns point to a complex, potentially treatment-sensitive relationship between *Erysipelotrichaceae* and mental health conditions. Therefore, the lower levels of *Erysipelotrichaceae UCG-003* in SCZ patients in our study may reflect disease-related dysbiosis or treatment effects, rather than a straightforward pathogenic role. Further studies are needed to determine whether this genus plays a protective, neutral, or detrimental role in the GBA axis of psychiatric disorders.

*Phascolarctobacterium* and *Catenibacterium*, along with *Holdemanella*, were among the differentially abundant genera identified in the psychiatric group, according to our LEfSe analysis. Notably,* Phascolarctobacterium* has previously been reported as enriched in SCZ patients [100, 124]. The species *Phascolarctobacterium succinatutens* metabolizes succinate [125, 126] to produce SCFAs such as propionic and acetic acids [127], whereas *Catenibacterium* appears to be more abundant in other neuropsychiatric disorders, including moderate MDD [128], posttraumatic stress disorder (PTSD) [129], and in the oropharynx of the SCZ patients [130]. Results on diversity metrics, both inter- and intra-specific have indicated a clear difference between SCZ and HCs, associated with a higher prevalence of *Ruminococcaceae* and lactic bacteria, which relate to worse clinical outcomes [131]. Mapping the gut microbiome in SCZ and various mental illnesses is crucial for identifying transdiagnostic markers, which may be indicated by reduced levels of SCFAs-producing genera [55], depleted levels of *Faecalibacterium* and *Coprococcus*, and enriched *Eggerthella* [132].

Taken together, our analyses demonstrate that SCZ status is a robust driver of gut microbial differences even after adjusting for demographic, metabolic, lifestyle, and comorbidity factors. Within the SCZ cohort, risperidone was the only medication significantly associated with β-diversity. This finding aligns with a previous study that shows risperidone uniquely downregulates serotonin 2A receptors (5-HT_2_A) and the serotonin transporter (SERT) in peripheral blood mononuclear cells when compared to other antipsychotics [133] with likely parallels in the gut epithelium. Since over 90% of the body’s serotonin is produced in the intestine and modulates motility, barrier integrity, and mucosal immunity [134], risperidone’s serotonergic modulation may remodel the intestinal niche in favor of specific taxa. Elucidating which microbes are most affected will require larger cohorts, but our results underscore the need to account for psychotropic-induced microbiome shifts when choosing antipsychotic therapies. Adjuvant interventions such as targeted probiotics or dietary modulation could serve as potential strategies to counteract the downstream metabolic or inflammatory consequences of antipsychotics.

### Limitations and highlights of the study

The study has several limitations that should be acknowledged. The most significant is the relatively small sample size, which, as in similar studies, limits the generalizability of the findings to populations beyond those studied. While our study achieved sufficient power to detect large taxon-level shifts, as demonstrated by our post-hoc power analysis on the top 3 most differentially abundant genera between groups, it remains underpowered for detecting small to medium effects. In fact, detecting a medium effect (Cohen’s *d* ≈ 0.5) would require approximately 64 subjects per group. Consequently, while our current design is adequately powered for the robust, large shifts reported here, future studies with larger cohorts are necessary to uncover more subtle microbial differences and to enhance the generalizability of our findings. As such, the results should be interpreted with caution, particularly regarding their applicability to other regions and ethnic groups. Additionally, the groups were not matched 1:1 due to the method used to control for confounders, and only one stool sample per individual was collected. This also limits the extrapolation to the broader population of patients with SCZ, as gut microbiome composition can vary over time and be influenced by many factors [135, 136], reflected by an intra-individual variability greater than between-group variability [137]. Moreover, SCZ patients differed in their medication regimens and treatment durations, and since medication can affect both microbiota composition and brain activity, this may have influenced the results. All participants were hospitalized and received standardized, hospital-provided diets, which may not reflect the broader SCZ population. Given these aspects, future research should involve larger, more diverse cohorts to validate and generalize these findings. Additionally, the absence of metabolomic analyses to correlate SCFAs with microbial composition, the lack of inflammatory markers, and the fact that this was a pilot rather than a clinical trial further highlight the need for more comprehensive studies.

## Conclusions

In summary, our study revealed distinct SCZ and HC gut microbiome profiles. In particular, SCZ is characterized by decreased *Bifidobacterium*, *Blautia*, and *Eubacterium* abundances, alongside enrichments of *Holdemanella*, *Phascolarctobacterium* and *Catenibacterium*. sPLS-DA further identified *Anaerostipes* and *Erysipelotrichaceae UCG-003* as key principal drivers of HC group clustering, and *Holdemanella*, as the primary discriminator of SCZ. Our findings potentially suggest diminished SCFA levels in SCZ and altered gut-brain signaling which may be exacerbated by risperidone treatment. These results underscore the potential of microbiome-based interventions in SCZ and highlight the urgent need for region-specific studies in Romania and neighboring Eastern European countries, where such analyses are currently lacking.

## Data Availability

The raw demultiplexed sequences analyzed in this study and corresponding metadata are deposited in Zenodo (https://zenodo.org/records/13353159), DOI 10.5281/zenodo.13353158. The code for sequence processing, statistical analyses and figure generation can be obtained by reasonable request from the corresponding author.

## Abbreviations

SCZ: Schizophrenia
GI: Gastrointestinal
GBA: Gut–brain axis
HCs: Healthy controls
PCoA: Principal coordinate analysis
PERMANOVA: Permutational multivariate analysis of variance
CLR: Centered log–ratio
PCA: Principal component analysis
sPLS-DA: Sparse partial least squares linear discriminant analysis
FDR: False discovery rate
LEfSe: Linear discriminant analysis (LDA) effect size
GBD: Global Burden of Disease Study
YLD: Years lived with disability
DALY: Disability–adjusted life years
EU: European Union
WHO: World Health Organization
QoL: Quality of life
CNS: Central nervous system
GABA: Gamma–aminobutyric acid
SCFA: Short–chain fatty acid
BBB: Blood–brain barrier
SGAs: Second–generation antipsychotics
ICD-10: International Classification of Diseases–10
BPRS: Brief Psychiatric Rating Scale
gDNA: genomic DNA
ASVs: Amplicon sequence variants
Faith’s PD: Faith’s phylogenetical distance
VST: Variance stabilizing transformation
BH: Benjamini–Hichberg
VIF: Variance inflation factor
PC1: Principal component 1
SANS: Scale for the Assessment of Negative Symptoms
ASD: Autism spectrum disorder
AD: Alzheimer’s disease
FGID: Functional gastrointestinal disorders
CD: Crohn’s disease
MDD: Major depressive disorder
AICP: Alcohol–induced chronic pancreatitis
PTSD: Posttraumatic stress disorder
5-HT2A: Serotonin 2A receptor
SERT: Serotonin transporter
